# Is there an association between dental caries, fluorosis, and molar-incisor hypomineralization?

**DOI:** 10.1590/1678-7757-2020-0890

**Published:** 2021-07-16

**Authors:** Marília Bizinoto Silva Duarte, Vanessa Reinaldo Carvalho, Leandro Augusto Hilgert, Ana Paula Dias Ribeiro, Soraya Coelho Leal, Eliana Mitsue Takeshita

**Affiliations:** 1 Universidade de Brasília Faculdade de Ciências da Saúde Departamento de Odontologia Distrito Federal Brasil Universidade de Brasília, Faculdade de Ciências da Saúde, Departamento de Odontologia, Distrito Federal, Brasil; 2 University of Florida Department of Restorative Dental Sciences GainesvilleFL USA University of Florida, Department of Restorative Dental Sciences, Gainesville, FL, USA.

**Keywords:** Molar-incisor hypomineralization, MIH, Dental fluorosis, Dental caries

## Abstract

**Objective:**

This cross-sectional study aimed to determine the prevalence of dental caries, dental fluorosis, and molar-incisor hypomineralization, and their associations in a group of Brazilian schoolchildren.

**Methodology:**

Adolescents (n=411) were evaluated by two calibrated examiners for dental caries (DC), dental fluorosis (DF), and molar-incisor hypomineralization (MIH) using the CAST (Caries Assessment Spectrum and Treatment) instrument, Thylstrup and Fejerskov (TF) index, and MIH Severity Scoring System (MIH-SSS), respectively. Descriptive statistics, chi-square tests, and logistic regression were used for statistical analysis.

**Results:**

The sample comprised 42.75% boys and 57.25% girls. The prevalence of DC in permanent dentition was 94.75%, of which 29% were represented by dentin lesions. For DF, a prevalence of 40.75% was observed, with 69.32% mild, 12.88% moderate, and 17.79% severe. A positive association between the source of water and fluorosis was detected (p=0.01). The prevalence of MIH was 18%. Thirty adolescents (41.7%) presented with severe MIH. No association was found between DF or MIH and dentin DC or between MIH and DF at the individual level. However, a significant negative relationship was detected between DF and dentin carious lesions ( *p* <0.005) and DF and MIH ( *p* <0.00001) at the tooth level, whereas a positive association was observed between MIH and dentin carious lesions ( *p* <0.00001). A positive association was also observed between the severity of both conditions ( *p* <0.00001). Mild DF was the most prevalent problem observed. Cases of teeth with mild MIH were the most predominant in MIH-affected teeth.

**Conclusions:**

No association was observed among the dentin carious lesions, MIH, and DF at the participant level. However, a positive association between MIH and dentin carious lesions was found at the tooth level, whereas MIH, DF, and DF and dentin carious lesions showed a negative relationship.

## Introduction

Tooth structures can be damaged before and/or after tooth eruption. Before eruption, disturbances to ameloblasts during amelogenesis may affect the appearance and structure of the enamel of both primary and permanent teeth, an event described as developmental defects of the enamel (DDE). ^1^^,^^2^ After eruption, the most frequent problem affecting the integrity of children's teeth is dental caries (DC). ^3^ Moreover, both dental caries and DDEs can be observed on the same tooth, with evidence showing that DDEs may be a risk factor for dental caries onset. ^4^^,^^5^

DDEs are classified into qualitative and quantitative defects. Qualitative defects result from an alteration during the enamel mineralization process. This is observed as opacities that can be demarcated or diffuse and can vary in color, from white/yellow to brown, without enamel thickness impairment. ^2^ On the other hand, quantitative defects are a consequence of an inappropriate deposition of the organic matrix, producing hypoplastic areas in which the enamel was not formed; this absence of enamel can lead to morphological and functional impairment of the affected teeth. ^1^^,^^2^^,^^6^^,^^7^

Considering the prevalence of DDEs, qualitative defects are more prevalent than quantitative defects. This includes dental fluorosis (DF) and molar-incisor hypomineralization (MIH). ^2^^,^^7^ However, both conditions present different clinical features: the opacities observed in DF are diffused and those in MIH are demarcated. A correct diagnosis of DF and MIH may be challenging when the same teeth are affected. Furthermore, both problems may be associated with some esthetic dissatisfaction, especially the anterior teeth. ^8^^,^^9^

Depending on the type and severity of the DDE, it can sometimes go undetected. However, it can negatively affect the quality of life of some individuals ^7^ due to esthetic problems, sensitivity, greater susceptibility to dental caries, and even an increased risk of tooth loss. ^1^^,^^7^^,^^10^

The diagnosis of DDEs is considered difficult and may be confused with dental caries. Therefore, dentists have been recommended to record both conditions during clinical examination. ^1^ It is not uncommon, for example, that severe cases of MIH, in which post-eruptive breakdown has occurred, are misdiagnosed as carious lesions. This is relevant because the longer the teeth are in the oral cavity, the more susceptible they are to post-eruptive breakdown. ^11^^,^^12^ However, information about the oral health condition of individuals affected by MIH when they grow older is sparse, since prevalence studies are typically conducted in children from 6 to 10 years of age. ^13^ Additionally, identification of DF may be more straightforward in adolescents than in children. This is because they have more permanent teeth, especially homologous teeth, thus contributing to a more reliable diagnosis of fluorosis. ^14^

To our knowledge, only two studies have investigated the correlation between DF, DC, and MIH. They reported an association between MIH and DC and between MIH and DF. ^11^^,^^15^ However, the diagnostic indexes used in both studies to classify all conditions are not the most discriminative. Additionally, the 7 to 9 years age group in one of the studies was not ideal for DF diagnosis. Therefore, this study aimed to investigate the prevalence, severity, and possible association between MIH, DF, and DC in a population of adolescents (11 to 14 years old), since there is a lack of information in this Brazilian population.

## Methodology

### Study design and ethical aspects

This cross-sectional study was approved by the Human Research Ethics Committee of the Faculty of Health Sciences, University of Brasília, under the number CAAE 63889716.6.0000.0030 and by the Secretary of Education of the Federal District. Parents and adolescents signed informed consent and assent forms, respectively, before the examination.

Paranoá (Human Development Index – HDI=0.785), the area in which this study was conducted, is one of the 33 administrative regions of the Federal District. According to the most recent official governmental data, it has a population of approximately 61,000 inhabitants, of which 89.1% reported not having health insurance.

### Sample size calculation and study participants

The sample size was estimated based on the formula $\text{n}=\left[\text{EDFF}*\text{Np}(1-\text{p})\right]/[\left({\text{d}}^{2}/{\text{Z}}_{1-\text{a}/2}^{2}*(\text{N}-1)+\text{p}*(1-\text{p})\right]$ , in which the population size is represented by (N), the frequency in the hypothetical percentage of the resulting factor in the population is (p), the confidence limits are absolute 100% (+/-%) (d), the standard score of normal distribution (Z), and the design effect is (EDFF). A population of 4,300, which corresponds to the total number of schoolchildren between 11 and 14 years old enrolled in the schools of Paranoá, Brazil, was considered. Two calculations were performed considering the prevalence of 48.5% ^16^ for DF and 14.69% ^17^ for MIH. The results indicated a minimum of 353 adolescents for DF and 185 adolescents for MIH.

The inclusion criteria were healthy children aged between 11 and 14 years. Children 1) with dental hypersensitivity and enamel defects other than MIH and DF, 2) under orthodontic treatment, 3) with special needs that would impair proper examination, and 4) whose parents did not sign the informed consent were excluded from the analysis.

The two largest schools located in Paranoá, out of the five schools enrolling students between 11 and 14 years old, were selected. All students in the aforementioned age range from both schools were invited to participate. Four hundred and eleven students returned with the informed consent signed by their parents. Clinical examinations were performed in 2017. Of the 411 adolescents examined, 11 had their data excluded based on two exclusion criteria: use of orthodontic appliances and amelogenesis imperfecta.

### Examiners calibration

Two examiners were trained and calibrated on the Caries Assessment Spectrum and Treatment (CAST) instrument, ^18^ on the Thylstrup and Fejerskov (TF) index, ^14^ and on the MIH-SSS (MIH Severity Scoring System), ^12^ used to record DC, DF, and MIH, respectively. The training started with a 4-hour theoretical lecture that covered DC and both enamel defects, and characteristics of each diagnostic criterion. Subsequently, a series of images were used following the *in lux* calibration for DF and MIH. Finally, for DC, an *in vitro* calibration was performed on the extracted teeth.

One week after, the examiners performed 8 hours of clinical calibration to assess children of the same age as those who would be included in the main study. Within another seven-day interval, the same clinical exercise was performed under field conditions. In both activities, the examiners were supervised by two senior investigators that were familiar with the diagnostic criteria. The intra- and interexaminer kappa values were 0.64, 0.61, and 0.66 for CAST, 0.83, 0.78, and 0.88 for MIH-SSS, 0.65, 0.70 and 0.67 for TF index, respectively.

### Clinical examination and Socio-demographic questionnaire

Examinations were performed indoors, on the school premises, by the two trained and calibrated examiners, following the order: dental caries, MIH, and DF detection. Prior to the clinical examination, the plaque was mechanically removed with a toothbrush without toothpaste, and excess saliva was removed with gauze. A portable bed, headlamps (LL 82001, China) as indirect light, clinical mirrors no. 5, and WHO millimeter probes (Millenniun Golgran, São Paulo, Brazil) were used. Data were recorded in a specific form by two trained note-takers. For DC and MIH, every tooth surface was recorded, with the most severe score registered in case of two or more defects on the same surface, and tabulated using CAST and MIH-SSS, respectively. For DF, the tooth was used as a unit to record the presence and severity using the TF index.

A sociodemographic questionnaire with questions on the use of fluoridated toothpaste, brushing times per day, swallowing of toothpaste during infancy, and the source of drinking water was answered by the parents after clinical examination by phone interview.

### Data analysis

The variables MIH, DF, and dentin carious lesions were categorized as “present” or “absent.” MIH was categorized according to severity as mild (MIH-SSS 1 and 2=demarcated opacities), moderate (MIH-SSS 3=post-eruptive breakdown restricted to enamel), and severe (MIH-SSS 4 or greater = post-eruptive breakdown involving dentin, atypical restoration, and tooth loss due to MIH). DF was also categorized according to severity as mild (TF 1 and 2), moderate (TF 3 and 4), and severe (TF 5 or greater). Dentin carious lesions were classified as “present” (CAST codes 4-7) or “absent” (CAST codes 0–3).

The examined population presented a late mixed dentition, and data from primary and permanent dentitions were presented separately (336 and 10,455 deciduous and permanent teeth, respectively). Although the study population presented with mixed dentition, only the permanent teeth were considered for the evaluation of the association between the three conditions on the tooth level. For the individual-level analysis, it was observed that all children with DC in the primary dentition also presented with DC in the permanent dentition. In total, 10,455 teeth (only permanent dentition) were used for DC and DF analysis. Meanwhile, only the first permanent molars and permanent incisors (central and lateral) were considered for MIH (4,780 teeth).

Bivariate analysis using Chi-square test (χ²) with a 5% significance level was used to evaluate the associations among the three conditions. For the tooth level analysis, a logistic regression model was used to obtain the odds ratio using the independent variables with p<0.20. In the bivariate analysis, a model according to stepwise forward selection was considered. The software used was Stata / SE 15.1 (StataCorp, College Station, TX 77845, USA).

## Results

In total, 400 adolescents, 171 boys and 229 girls with a mean age of 12 ±1 years, were examined (234 from school 1 and 166 from school 2), and 291 parents responded to the sociodemographic questionnaire (response rate of 72.75%).

Concerning the sociodemographic information, approximately 56% of the mothers worked outside the home and, most of them had not completed elementary/middle school (35%), 11% had not completed high school, and 26% had completed high school. Regarding the father's educational level, most of them also had not completed elementary/middle school (34%), 9% had not completed high school, and only 17% had completed high school. Most families lived in houses (56%) that were rented (32%) and had a monthly income of up to one (48%) or between 1 and 2 (38%) Brazilian minimum wages (approximately 200 US dollars). Most of the families had only one family member that works (64%). The mean number of people living in the same household was around 5±2.

**Figure 1:**
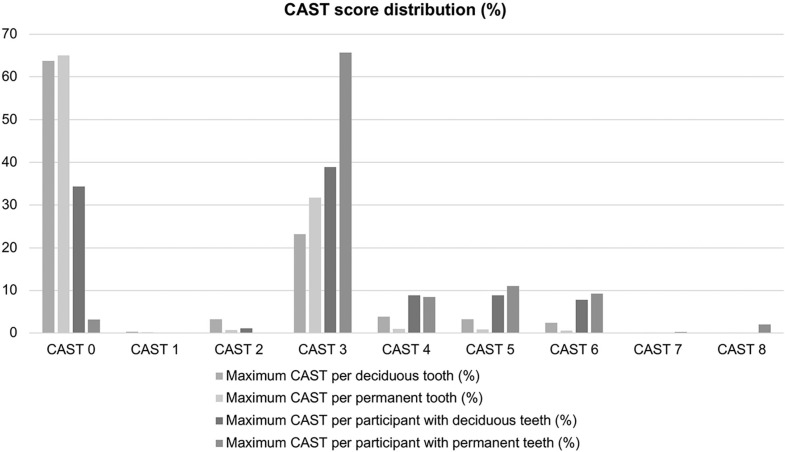
Bar chart with the percentage of CAST scores for deciduous and permanent dentition both at the tooth and at the individual levels

For dental caries, the majority of teeth, whether primary or permanent, were classified as sound (CAST 0). However, when the maximum CAST per participant was estimated, for the permanent dentition, 94.75% presented DC (CAST 3 to 7). Considering the enamel (CAST 3) or dentin carious lesions (CAST 4, 5, 6, and 7), approximately 66% and 29% of the sample had at least one of these types of lesions, respectively. Figure 1 presents the CAST scores percentage for deciduous and permanent dentition considering teeth and individual levels.

For DF, 10,455 teeth (400 adolescents) were evaluated. Table 1 shows the distribution of the adolescents according to the TF index and for severity. A prevalence of 40.75% (163 adolescents) was observed, with mild cases (TF 1 and 2) being the most prevalent. No association was observed between DF and gender (χ², *p* =0.92) or fluorosis and dentin carious lesions (CAST codes 4-7) (χ², *p* =0.24). Table 2 shows the distribution of adolescents according to the variables related to fluoride sources. A significant difference was observed for the water source. A greater number of adolescents without fluorosis ingested well water than adolescents with fluorosis (χ², *p* =0.01).

**Table 1:** Distribution of teeth and participants according to the levels of fluorosis considering the TF index

TF Score	TF per tooth (%)	Maximum TF per participant (%)
TF 0	8504 (81.34)	237 (59.25)
TF 1	1316 (12.58)	56 (14.00)
TF 2	392 (3.75)	57 (14.25)
TF 3	101 (0.97)	15 (3.75)
TF 4	15 (0.14)	6 (1.50)
TF 5	118 (1.13)	23 (5.75)
TF 6	6 (0.06)	3 (0.75)
TF 7	3 (0.03)	3 (0.75)

**Table 2:** Distribution of adolescents with and without fluorosis according to the information retrieved from the questionnaire related to the use of fluoride

Questions	Adolescents without fluorosis	Adolescents with fluorosis	p-value *
Toothpaste with or without fluoride	With=137	With=100	0.376
	Without=33	Without=19	
	Did not know=2		
Brushing per day	One=30	One=18	0.602
	Two=66	Two=52	
	Three=69	Three=42	
	Over three=6	Over three=7	
	Did not know=1		
Toothpaste intake	Yes=97	Yes=76	0.33
	No=74	No=43	
	Did not know=1		
Drinking water source	Public=145	Public=95	0.001
	Bottled=7	Bottled=19	
	Artesian well=19	Artesian well=5	
	Did not know=1		

* χ² statistical test

Regarding MIH, a prevalence of 18% was detected, affecting 72 adolescents and 227 teeth. From this total, 36 children (50%) were classified as mild, 6 (8.3%) as moderate, and 30 (41.7%) as severe.

When the three conditions (DC, MIH, and DF) were evaluated per adolescent, dentin carious lesions, MIH, and DF coexisted in about 2.5% of the adolescents. Table 3 shows the distribution of adolescents according to the three conditions.

**Table 3:** Distribution of participants according to the three dental conditions evaluated

Dental condition	Frequency (%)
Adolescents without any dental condition	132 (33.00)
Dentin carious lesions only	61 (15.25)
Fluorosis only	101 (25.25)
MIH only	28 (7.00)
Dentin carious lesions + fluorosis	34 (8.50)
Dentin carious lesions + MIH	16 (4.00)
Fluorosis + MIH	18 (4.00)
Dentin carious lesions + fluorosis + MIH	10 (2.50)

At the individual level, there was no association between the maximum CAST score and the presence of fluorosis (Fisher's test, *p* =0.59) or MIH (Fisher's test, *p* =0.26). The association between the presence of dentin carious lesions (CAST 4 to 7) and fluorosis was not observed (χ², *p* =0.24; Table 4 ). Of the 163 children with fluorosis, 119 had no dentin carious lesions, whereas 44 presented dentin carious lesions. For MIH, of the 72 children with MIH, 26 had dentin lesions (36.11%). However, no association was observed between the presence of MIH and carious lesions in the dentin (CAST 4 to 7) (χ², *p* =0.232; Table 4 ). When the association between fluorosis and MIH was assessed, there was also no association between these conditions (χ², *p* =0.939; Table 4 ). No association between the severities of the two conditions was observed (χ², *p* =0.25).

**Table 4:** Frequency and statistical analysis of the three dental conditions (DC, DF, MIH) at the individual level

	Dentin Lesions			
		*Absent*	*Present*	*p* -value *
**MIH**	*Absent*	233	95	
	*Present*	46	26	0.232
**Fluorosis**	*Absent*	160	77	
	*Present*	119	44	0.24
	**MIH**			
		Absent	Present	
**Fluorosis**	*Absent*	193	136	
	*Present*	42	29	0.939

* χ² statistical test

At the tooth level, 1,951 teeth presented DF; 1,708 were mild, 116 were moderate, and 127 were severe. Considering dentin carious lesions, 242 teeth were classified as CAST 4 to 7. When the relationship between dentin carious lesions and DF was evaluated considering the tooth as the unit of measurement, a significant negative association was observed between the two variables (χ², p<0.00001). Of the 1,951 teeth with fluorosis, only 20 of them presented dentin carious lesions ( Table 5 ). Moreover, no relation between the severity of fluorosis and the dentin lesion (χ², p>0.05) was observed.

**Table 5:** Frequency and statistical analysis of the three dental conditions (DC, DF, MIH) at the tooth level

	Dentin Lesions			
		*Absent*	*Present*	p-value*
**MIH**	*Absent*	4422	131	
	*Present*	194	33	< 0.00001
**Fluorosis**	*Absent*	8282	222	
	*Present*	1931	20	< 0.00001
	***MIH***			
		*Absent*	*Present*	
**Fluorosis**	*Absent*	3773	210	
	*Present*	780	17	< 0.00001

* χ² statistical test

Considering MIH, 227 teeth had MIH; 165 had mild, 10 had moderate, and 52 had severe MIH. Table 5 shows that a significant positive relationship between the MIH and dentin carious lesions was detected at the tooth level was detected (χ², *p* <0.00001). Furthermore, there was a significant correlation between dentin carious lesions and MIH severity. Of the 227 teeth classified with MIH, 33 presented dentin carious lesions, 28 of which were classified as having severe MIH (χ², *p* <0.00001).

Finally, when considering the relationship between MIH and DF, we observed a significant negative association between these two variables (χ², *p* <0.00001). Of the 227 teeth with MIH, only 17 teeth presented DF. No association was observed between the severity of MIH and the severity of fluorosis ( *p* =0.098).

A logistic regression model was used to obtain the odds ratio for dentin carious lesions in the presence of MIH and fluorosis. The results showed that teeth with severe MIH had a 5.4 times greater chance of having dentin carious lesions ( *p<* 0.0001; 95%CI, 3.57–8.10). Teeth with DF, in turn, were 0.35 times more likely to have dentin carious lesions, that is, 65% less likely to have dentin carious lesions ( *p=* 0.001; 95%CI, 0.18–0.67).

## Discussion

Our study evaluated the presence and severity of DC, DF, and MIH and their association in a group of socially vulnerable adolescents aged 11 to 14 years. We found an association among the three conditions; however, we decided to also report the findings of each condition separately. The population's socioeconomic background can partially explain the high prevalence of caries reported in a community with access to fluoridated water. When the variables “parental level of education” and “family income” were analyzed together, we learned that the adolescents investigated came from poor families. Besides, previous investigations in the same region have reported that the affordability of dental care in that locality is limited, ^19^ which also contributed to the high level of participants with untreated carious lesions.

Regarding DC detection, the CAST instrument used in our study is considered a suitable system for caries assessment in epidemiological surveys. ^20^ This takes only one minute longer to be performed than the DMF/dmf index. ^21^ Moreover, CAST provides a more detailed evaluation, since it includes the recordings of the enamel lesions. By using this strategy, we could identify a worrying scenario for permanent dentition: less than 4% of the adolescents were caries-free and approximately 66% presented at least one carious enamel lesion and 29% had dentinal caries. This emphasizes the importance of preventive dentistry and restorative treatment of dentin lesions in this population. Furthermore, the only treatment observed was extraction, since no sealed or restored teeth were observed. These clinical data contradict the information obtained in the sociodemographic questionnaire answered by the parents, in which the most reported that their children brushed their teeth three times a day with fluoridated toothpaste. This finding indicates that oral hygiene procedures were not being effectively performed and this draws attention to something already pointed out in the literature: knowledge and behavioral changes in oral health need to be improved for this age group. ^3^

Regarding DF, the fact that we included adolescents aged from 11 to 14 years was considered an advantage. It allowed for the evaluation of the anterior teeth that were fully erupted, contrary to other studies in which only the younger age groups were assessed, thus impeding an accurate diagnosis. The prevalence of DF was 40.75%, and the most prevalent cases were the mild cases, which are not considered a public health concern. ^16^ Both outcomes are consistent with previous studies, since the prevalence of DF varies from 16.7% to 65%, ^22^^,^^23^ and a predominance of mild cases is frequently reported. ^16^^,^^23^ However, 12.50% of the adolescents presented either moderate or severe DF, which can affect their quality of life. ^16^^,^^24^

DF severity is influenced by factors such as the amount of fluoride ingested, age and time of exposure, body weight, and some systemic conditions. ^14^^,^^24^ In our investigation, special attention was given to the sources of fluoride, as Paranoá is supplied with fluoridated water (0.6 to 0.8 mg/L) and most children reported using fluoridated toothpaste. According to our findings, the only variable associated with DF was the source of the water consumed. Adolescents that consumed water from artesian wells or bottled water in their childhood presented significantly less fluorosis than those who consumed public drinking water. This finding contradicts a systematic review that concluded that individuals exposed to artesian well water are at high risk of developing DF; however, it is important to emphasize that the comparison made in the review was between regions with non-fluoridated water and locations that used groundwater. ^24^ As mentioned, Paranoá's piped water contains fluoride, but the amount of fluoride in groundwater in the region is not known and was not measured, which limited the interpretation of our findings. Moreover, memory bias might have influenced the responses given by parents.

Regarding MIH, the prevalence found was 18%, similar to that of other studies in Brazil ^25^ and slightly higher than that reported globally (14.2%). ^13^ Differences in the examination methods, diagnostic criteria, and age of the participants can help to explain these variations. In our investigation, the decision to include adolescents (11–14 years old) is justified by the fact that there is a lack of information about the oral health status of participants in that age group. This is relevant, especially in a socially vulnerable population with a lack of access to dental care. One of the main concerns related to MIH is the post-eruptive breakdown over time, increasing the chances of tooth loss. ^12^

Our results found that 41.7% of the adolescents diagnosed with MIH already presented this condition at a severe stage, which was considered high. At the tooth level, 22.9% of the MIH-affected teeth were diagnosed as severe. This outcome was influenced by the patient's age, since the longer the affected enamel is exposed to masticatory forces, the greater the odds of damage to the tooth structure. ^7^^,^^26^ To our knowledge, only one study evaluated this specific age range, in which 10.75% of MIH-affected teeth were classified as severe. ^27^ However, an increased severity has been reported in children older than 10 years. ^25^^,^^26^ Since the definition of severe cases varied considerably among the studies, the results are difficult to compare. Both Costa-Silva, et al. ^25^ (2010) and Bhaskar and Hegde ^26^ (2014) included post-eruptive breakdown restricted to the enamel as severe cases, which is different from the study by de Lima, et al. ^27^ (2015) and the present investigation. However, if we compare our findings to a prevalence study conducted in the same region using the same diagnostic criteria (MIH-SSS), but assessing children aged 8 years, ^10^ a disturbing finding was observed: a dramatic increase in severe cases from 13% described by Raposo, et al. ^10^ (2019) to approximately 42% reported in our study. This finding reinforces the importance of early diagnosis of MIH followed by monitoring of the affected teeth.

Changes in enamel development are usually described in the literature. Our study is one of the first to present the association between MIH and DF showing specific indexes for each condition, in contrast to the modified DDE index, which allows the characterization of only diffuse or demarcated opacities. ^6^

When analyzing the association of the conditions at the participant level, it was observed that only 4% of the adolescents simultaneously presented MIH and DF. However, there was no association between the severities of the two conditions, in opposition to Fernandes, Fortes and Sampaio ^15^ (2020), who found an association between the severity of MIH and DF in areas with moderate to high fluoride levels in the drinking water. However, at the tooth level, an inverse significant association between both conditions was found in our study. Teeth with fluorosis were less likely to present with MIH, and, again, no severity association was observed between them. This finding is explained by the fact that DF and MIH have distinct etiologies and result from changes at different stages of enamel development ^28^ and that Paranoá is supplied with fluoridated water within the recommended levels. However, both conditions can coexist on the same tooth.

It is important to emphasize that the diagnosis of MIH does not condemn the tooth to develop DC. The assessment of an older group of participants than what is usually recommended ^6^^,^^13^ helped in the observation of the severity of MIH and its consequences. Previous studies showed a relationship between dental caries and MIH, ^5^^,^^29^ which corroborates our findings that showed a positive significant association between the two conditions at the tooth level. According to Negre-Barber, et al. ^30^ (2018) in areas with a high prevalence of caries, MIH can remain difficult to diagnose, since rapid caries progression eliminates any trace of this condition. Besides, the more severely the tooth is affected, the greater the chances of requiring restorative treatment and re-interventions throughout life, which can eventually lead to endodontic treatment or tooth loss prematurely. ^31^ Moreover, due to the less mineralized enamel, MIH is believed to increase tooth hypersensitivity in some patients, interfering with proper dental hygiene and thus increasing the risk of development of DC. ^10^

When analyzing the association between the DF and dentin caries lesions at the participant level, both conditions were present in only 8.5% of the adolescents, with no clinical significance. However, it has already been stated that studies based on tooth-level analysis are more relevant than those of the participant level, ^4^ which is reinforced by our findings, since an inverse relation between DF and DC was observed at the tooth-level, which is supported by Iida and Kumar ^4^ (2019). A possible explanation for the negative association between DF and DC is that higher concentrations of fluoride in the enamel make it more resistant to acid attack and can promote remineralization. ^4^ However, different findings have been described ^23^ and an *in vitro* study showed lower resistance of a fluorotic enamel to demineralization. ^32^ Considering that a fluorotic enamel is more porous, the fluoride from dentifrice used by the adolescents might have been able to diffuse throughout the enamel, protecting from demineralization. ^32^

Limitations of our study include the memory bias that might have affected the parents’ answers regarding the use of fluoride and the fact that no information about the amount of fluoride in the well water was available. Moreover, an eating and dietary habits survey was not performed, and, since sugar consumption directly influences the development of DC, the higher number of DCs found in this population could also be explained, at least in part, by their dietary patterns. ^3^ However, our study also presents important strengths, such as the inclusion of more than 300 participants for MIH, as recommended by Elfrink, et al. ^33^ (2015) and the application of detailed diagnostic criteria for all conditions investigated.

The high number of untreated DCs emphasizes the studied population's lack of access to dental care. Cases of teeth with mild MIH were the most predominant in MIH-affected teeth. DF was the most prevalent problem detected, whose cases were mostly mild. Further studies are necessary to estimate the prevalence and severities of these dental conditions and their associations, especially in areas where access to dental care is limited.

## Conclusion

No association between DC, MIH, and DF was observed at the participant level; however, at the tooth level, MIH was positively associated with dentin carious lesions, whereas MIH, DF, and DF and dentin carious lesions showed a negative association.
